# Single-cell RNA sequencing using split-pool barcoding reveals transcriptional heterogeneity in *Porphyromonas gingivalis* with implications for periodontal pathogenesis

**DOI:** 10.1080/20002297.2025.2540827

**Published:** 2025-07-31

**Authors:** Eun-Young Jang, Seok Bin Yang, Jeewan Chun, Kyu Hwan Kwack, Sang-Wook Kang, Jae-Hyung Lee, Ji-Hoi Moon

**Affiliations:** a Department of Oral Microbiology, College of Dentistry, Kyung Hee University, Seoul, Republic of Korea; b Department of Dentistry, Graduate School, Kyung Hee University, Seoul, Republic of Korea; c Department of Oral and Maxillofacial Pathology, College of Dentistry, Kyung Hee University, Seoul, Republic of Korea

**Keywords:** *Porphyromonas gingivalis*, single-cell analysis, RNA sequencing, bacterial gene expression, split-pool barcoding

## Abstract

**Background:**

*Porphyromonas gingivalis* is a keystone pathogen in periodontitis, associated with dysbiosis and chronic inflammation. While its virulence mechanisms are well characterized, its transcriptional heterogeneity at the single-cell level remains unexplored.

**Materials and Methods:**

We applied split-pool barcoding-based single-cell RNA sequencing to profile gene expression in 1,942 individual *P. gingivalis* W83 cells cultured under anaerobic conditions. Clustering and differential expression analyses were conducted to identify distinct transcriptional subpopulations.

**Results:**

We identified six transcriptionally distinct clusters, with the two largest accounting for 72.7% of the population. Minor clusters exhibited signatures related to stress responses, metabolism, membrane transport, and DNA regulation. Sub-clustering of major populations revealed rare subgroups, including one enriched for genes involved in iron acquisition, proteolysis, and transport.

**Conclusions:**

This study presents the first single-cell transcriptomic map of *P. gingivalis*, revealing rare but functionally significant subpopulations. Such diversity may support bacterial adaptability, virulence, and immune evasion, informing future strategies for targeted periodontal therapy.

## KEY MESSAGES

- Single-cell RNA sequencing reveals that *Porphyromonas gingivalis* comprises transcriptionally heterogeneous subpopulations, including rare subsets with distinct functional profiles.
- These functionally specialized subpopulations may promote bacterial persistence through environmental adaptation, immune evasion, and microbiome modulation.
- This study demonstrates that single-cell transcriptomics can uncover bacterial heterogeneity overlooked by bulk RNA-seq, providing new opportunities for targeted periodontal disease treatments.

## Introduction

*Porphyromonas gingivalis* is a non-motile, Gram-negative, black-pigmented anaerobe that plays a major role in the development and progression of chronic periodontitis. It utilizes various virulence factors, including fimbriae, proteolytic enzymes (gingipains), lipopolysaccharides, and outer membrane vesicles, to invade host tissues, evade immune responses, and disrupt microbial balance [1]. Beyond oral infections, *P. gingivalis* has also been implicated in systemic diseases such as cardiovascular disease and Alzheimer’s disease [1,2]. While its ability to survive and persist in different environments has been extensively studied at the population level, how individual bacterial cells within *P. gingivalis* communities dynamically regulate gene expression remains largely unknown. Given that bacterial populations often consist of functionally distinct subpopulations, capturing *P. gingivalis* heterogeneity at the single-cell level may provide insights into its virulence and adaptation strategies. For example, the presence of stress-tolerant or metabolically dormant subpopulations may help the bacterium persist in hostile environments such as the inflamed periodontal pocket, contributing to chronic infection and treatment resistance.

Single-cell transcriptomics, commonly referred to as single-cell RNA sequencing (scRNA-seq), has emerged as a powerful method to dissect this heterogeneity, revealing how individual cells within a population respond to environmental changes [3]. However, unlike eukaryotic cells, bacterial single-cell transcriptomics presents unique challenges, including low RNA content per cell, rigid cell walls, and rapid RNA degradation. These factors make it difficult to obtain high-quality data using conventional single-cell techniques [3].

To overcome these challenges, several single-cell sequencing methods have been developed, including split-pool barcoding – a scalable and efficient approach that is applicable even to prokaryotic cells. Split-pool barcoding, the method used in this study, allows high-throughput transcriptome profiling of thousands of cells without requiring physical isolation, reducing sample loss and contamination risks [4,5]. It is particularly advantageous for bacteria, which often have low RNA yields and complex cell walls, as it eliminates the need for extensive single-cell manipulation while ensuring scalability and reliability [3]. In this study, we applied PETRI-seq (Prokaryotic Expression-profiling by Tagging RNA In situ with sequencing), a bacterial-specific implementation of the split-pool barcoding strategy [4,5], to overcome these challenges.

Despite advancements in single-cell technologies, the transcriptional heterogeneity of *P. gingivalis* has remained unexplored. To fill this gap, we performed scRNA-seq using split-pool barcoding on *P. gingivalis* strain W83 to characterize its gene expression variability under optimal growth conditions. This reference strain is widely used in virulence and genomics studies due to its fully annotated genome and consistent phenotype across laboratories. Our approach represents the first application of bacterial single-cell transcriptomics to *P. gingivalis*, offering novel insights into how this keystone pathogen maintains phenotypic diversity within clonal populations. This diversity may underlie its ability to adapt to fluctuating host environments, evade immune surveillance, and persist during treatment, features that cannot be resolved using bulk RNA sequencing.

## Materials and methods

### Bacterial strains and growth conditions

*P. gingivalis* W83 was selected for the present study because its genome has been fully sequenced and extensively characterized in previous virulence studies, enabling accurate mapping and functional interpretation. The bacterium was routinely grown on either Tryptic soy agar (Becton, Dickinson and Company, USA) containing 5% laked sheep blood, 5 mg/L of hemin, and 1 mg/L of vitamin K_1_ (T-HK agar) or Brain heart infusion broth containing 5 mg/L of hemin and 1 mg/L of vitamin K_1_ (B-HK) at 37°C anaerobically (90% N_2_, 5% H_2_, 5% CO_2_). An overnight culture (10 mL) was grown to an optical density at 600 nm (OD_600_) of approximately 1.0–1.2, then diluted 1:100 into fresh B-HK medium (final volume: 30 mL) to initiate the subculture. Cells were harvested for PETRI-seq analysis when the subculture reached OD₆₀₀ = 0.35, corresponding to mid-exponential phase.

### Custom primers used in this study

All of the single-tube primers and the primer sequences for 96-well split-pool barcoding are shown in Table S1. Primers were purchased from Integrated DNA Technologies.

### Preparation of annealed ligation oligos

**Figure 1.**
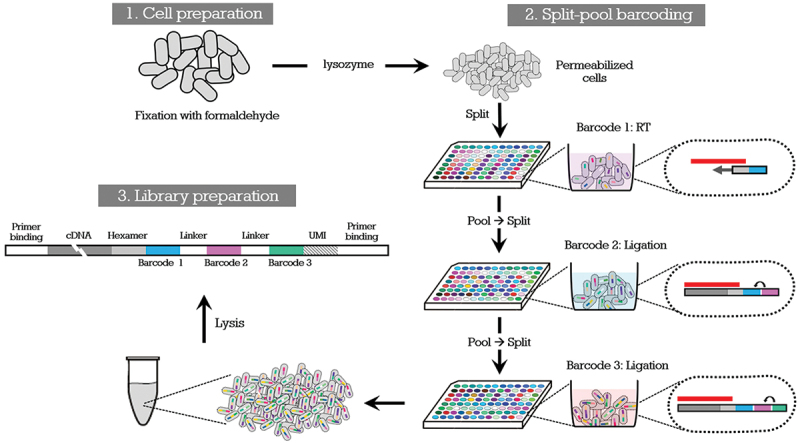
Schematic illustration of single-cell RNA sequencing using split-pool barcoding. This diagram, adapted from Moon et al (2023) [3], outlines the PETRI-seq workflow optimized for *P. gingivalis*. Bacterial single cells were fixed with formaldehyde and permeabilized with lysozyme. Split-pool barcoding was performed in three rounds: barcoded reverse transcription (RT), followed by two ligation steps introducing well-specific barcodes. This combinatorial indexing strategy ensures that transcripts originating from individual cells receive unique barcode combinations. After cell lysis, the resulting cDNA molecules contained three barcodes, a unique molecular identifier (UMI), and linker sequences for sequencing. The dotted oval represents a single bacterial cell, and the red horizontal bar symbolizes an individual RNA molecule within that cell. The sequences of the oligonucleotides used in this process are provided in Table S1.

The ligation primers used in round 2 and round 3 ligation reactions (Figure 1) were prepared by annealing barcode oligos to specific linker oligos (L2 and L3). Round 2 barcode oligos were diluted to 100 μM. Round 3 barcode oligos were diluted to 70 μM. Linker oligo L2 was diluted to 100 μM. Linker oligo L3 was diluted to 70 μM. To anneal Round 2 barcode oligos to linker oligos, a 96-well PCR plate (AB0600, Thermo Fisher Scientific) was prepared by adding 3.52 μL of diluted L2, 2.64 μL of water, and 3.84 μL of each round 2 barcode oligo to each well. To anneal round 3 barcode oligos, a 96-well PCR plate was prepared by adding 6.6 μL of diluted L3 and 7.2 μL of each round 3 barcode oligo to each well. Oligos were annealed by heating the plate to 95°C for 3 min and then decreasing the temperature to 20°C at a ramp speed of −0.1°C/s. Oligos B2–1 and B3–1 were also annealed (to form an intramolecular hairpin) before blocking by heating 50 μL or 80 μL, respectively, of each 400 μM oligo to 94°C and slowly decreasing the temperature to 25°C.

### Cell preparation for PETRI-seq

Cells were cultured overnight, then diluted in fresh medium and cultured until the OD_600_ reached 0.35. Before fixation, cells were centrifuged at 5,525 × g for 2 min at 4°C. The spent medium was removed, and cells were resuspended in 7 mL of ice-cold 4% formaldehyde in phosphate-buffered saline (PBS). The suspension was rotated on a shaker at 4°C for 16 h. The next day, fixed cells were centrifuged at 5,525 × g for 10 min at 4°C. The supernatant was removed, and the pellet was resuspended in 7 mL of PBS supplemented with 0.01 U/μL of SUPERase·In RNase Inhibitor (AM2696, Invitrogen), hereafter referred to as PBS-RI. Cells were centrifuged again at 5,525 × g for 10 min at 4°C and then resuspended in 700 μL of PBS-RI.

Subsequent centrifugations for cell preparation were all performed at 7,000 × g for 8–10 min at 4°C. Cells were centrifuged, then resuspended in 700 μL of 50% ethanol (E7023, Sigma-Aldrich) in PBS-RI. Cells were next washed twice with 700 μL of PBS-RI and resuspended in 105 μL of 100 μg/mL lysozyme (90082, Thermo Fisher Scientific) in TEL-RI [100 mM Tris, pH 8.0 (AM9856, Invitrogen); 50 mM EDTA (AM9261, Invitrogen); and 0.1 U/μL of SUPERase·In RNase Inhibitor (10× more than in PBS-RI)]. Cells were permeabilized for 15 min at approximately 23°C (room temperature). After permeabilization, cells were centrifuged, then washed with 175 μL of PBS-RI, and resuspended in 175 μL of PBS-RI. From this, 100 μL was taken for subsequent steps and centrifuged; the remaining 75 μL was discarded.

Cells were resuspended in 40 μL of DNase-RI buffer [4.4 μL of 10× reaction buffer, 0.2 μL of SUPERase·In RNase Inhibitor, and 35.4 μL of water]. Then, 4 μL of DNase I (AMPD1, Millipore Sigma) was added, and cells were incubated at room temperature for 30 min. To inactivate DNase I, 4 μL of stop solution was added, and the cells were heated to 50°C for 10 min with shaking at 500 rpm (Note: 50°C, rather than 70°C, was used to avoid cell lysis). After DNase inactivation, cells were pelleted, washed twice with 100 μL of PBS-RI, and then resuspended in 100 μL of 0.5× PBS-RI. Cells were counted using a hemocytometer (DHC-S02, INCYTO).

### Split-pool barcoding

For reverse transcription (RT), round 1 primers (Table S2) were diluted to 10 μM, then 2 μL of each primer was aliquoted into a 96-well PCR plate. The RT reaction mix was prepared using 240 μL of 5× RT buffer, 24 μL of dNTPs (N0447L, NEB), 12 μL of SUPERase·In RNase Inhibitor, and 24 μL of Maxima H Minus Reverse Transcriptase (EP0753, Thermo Fisher Scientific). A total of 3 × 10^7^ cells were added to this mix. Nuclease-free water was added to bring the volume to 960 μL. Then 8 μL of the reaction mixture was added to each well of the 96-well plate containing the RT primers, bringing the final volume in each well 10 μL. The plate was sealed and incubated under the following thermal conditions: 50°C for 10 min; 8°C for 12 s; 15°C for 45 s; 20°C for 45 s; 30°C for 30 s; 42°C for 6 min; 50°C for 16 min; and then held at 4°C.

After RT, the 96 reactions were pooled into a single tube. To permeabilize the pooled cells, detergent was added as follows: 5% Tween-20 was diluted 1:125 to reach a final concentration of 0.04%. We measured the volume of the pooled cells to determine this exact volume. Cells were incubated on ice for 3 min, then PBS-RI was added to reduce the final concentration of Tween-20 to 0.01%. Cells were then centrifuged at 10,000 × g for 20 min at 4°C, and the supernatant was carefully removed.

For the round 2 ligation, cells were resuspended in 600 μL of 1× T4 ligase buffer (M0202L, NEB) supplemented with 0.1 U/μL of SUPERase·In RNase Inhibitor. To prepare the ligation master mix, the following components were combined: 7.5 μL of nuclease-free water, 37.5 μL of 10× T4 ligase buffer, 16.7 μL of SUPERase·In RNase Inhibitor, 5.6 μL of BSA (B14, Thermo Fisher Scientific), and 27.9 μL of T4 ligase, yielding a total volume of 695.2 μL. From this mix, 5.76 μL was added to each well of a 96-well plate already containing 2.24 μL of annealed round 2 ligation oligos, resulting in a final reaction volume of 8 μL. Ligation reactions were incubated at 37°C for 30 min. Following this incubation, 2 μL of blocking mix was added to each well. The blocking mix was prepared by combining 37.5 μL of 400 μM B2–1 oligo, 37.5 μL of 400 μM B2–2 oligo, 25 μL of 10× T4 ligase buffer, and 150 μL of nuclease-free water. The plate was then incubated at 37°C for an additional 30 min. After completion of the ligation and blocking steps, the reactions were pooled into a single tube for subsequent processing.

For round 3 barcoding, the following reagents were added to the pooled cells: 46 μL of 10× T4 ligase buffer, 12.65 μL of T4 ligase, and 115 μL of nuclease-free water. From this mixture, 8.51 μL was added to each well of a 96-well plate already containing 3.49 μL of annealed round 3 ligation oligos. The plate was incubated at 37°C for 30 min. After ligation, 10 μL of round 3 blocking mix was added to each well. The blocking mix consisted of 72 μL of 400 μM B3–1 oligo, 72 μL of 400 μM B3–2 oligo, 120 μL of 10× T4 ligase buffer, 336 μL of nuclease-free water, and 600 μL of 0.5 M EDTA. Following this incubation, cells were pooled into a single tube. Tween-20 was then added to the pooled cells to a final concentration of 0.01%. Cells were centrifuged at 7,000 × g for 10 min at 4°C and resuspended in 500 μL of TEL-RI supplemented with 0.01% Tween-20. This suspension was centrifuged again under the same conditions, and the supernatant was removed. Cells were then resuspended in 30 μL of TEL-RI buffer and counted using a haemocytometer. Aliquots of approximately 1.6 × 10^6^ cells were mixed with 25 μL of 2× lysis buffer [containing 50 mM EDTA, 400 mM NaCl (AM9759, Invitrogen) and 1% Triton X-100]. Proteinase K (5 μL of 20 mg/mL; AM2548, Invitrogen) was then added to the mixture. Cell lysis was performed by incubating the samples at 55°C for 1 h with shaking at 750 rpm (Multi-Therm). Lysates were stored at −80°C until further use.

### Library preparation

Library preparation steps following cell lysis and preceding PCR amplification must be performed with particular care, as each barcoded cDNA molecule derived from a single cell is irreplaceable once lost. In other words, any loss of cDNA directly reduces the number of captured unique molecular identifiers (UMIs) per cell. Cell lysates were purified using 1.8× volume (approximately 99 μL) of AMPure XP beads (A63881, Beckman Coulter), and cDNA was eluted in 20 μL of nuclease-free water. To the eluted cDNA, 14 μL of nuclease-free water, 4 μL of NEBNext Second Strand Synthesis Reaction Buffer, and 2 μL of NEBNext Second Strand Synthesis Enzyme Mix (E6111S, NEB) were added, bringing the total reaction volume to 40 μL. The mixture was incubated at 16°C for 2.5 h to synthesize double-stranded cDNA. The resulting double-stranded cDNA was purified again using AMPure XP beads at 1.8× ratio (approximately 72 μL of beads) and in 20 μL of nuclease-free water. The purified cDNA was either immediately used for tagmentation or stored at −20°C. Tagmentation and subsequent amplification of double-stranded cDNA were performed using the Nextera XT DNA Library Preparation Kit (FC-131–1096, Illumina).

While we generally followed the manufacturer’s protocol, we applied the following modifications to reagent volumes and primers. For tagmentation, 25 μL of Tagment DNA (TD) buffer and 5 μL of Amplicon Tagment Mix (ATM) enzyme were added to the purified lysate, followed by a brief centrifugation. The reaction was incubated on a preprogrammed thermocycler at 55°C for 5 min and then held at 10°C. Once the reaction reached 10°C, 12.5 μL of Neutralize Tagment (NT) buffer was immediately added, followed by another brief spin-down and incubation at room temperature for 5 min.

For PCR amplification, a master mix consisting of 2.5 μL of i50x primer (E7600S, NEB), 2.5 μL of N70x primer (Nextera Index Kit v2 Set A, TG-131–2001, Illumina), 20 μL of nuclease-free water, and 37.5 μL of Nextera PCR Master Mix (NPM) was added to the reaction. Libraries were amplified using the following thermocycling conditions: initial gap-filling at 72°C for 3 min, followed by denaturation at 95°C for 30 s. This was followed by 16 cycles of 95°C for 10 s (denaturation), 55°C for 30 s (annealing), and 72°C for 30 s (extension). A final extension was performed at 72°C for 5 min, and the reaction was then held at 4°C. Amplified libraries were purified using AMPure XP beads at a 1× ratio and eluted in 30 μL of nuclease-free water. Library concentration and quality were assessed using the Qubit dsDNA High Sensitivity Assay Kit (Q32854, Invitrogen) and the High Sensitivity DNA Kit for the Agilent Bioanalyzer (5067–4626, Agilent Technologies). Sequencing was performed using the NextSeq 500/550 High Output Kit v2.5 (75 cycles; 20024906, Illumina), with cycle allocation as follows: 58 cycles for Read 1 (UMI and barcodes), 17 cycles Read 2 (cDNA), 8 cycles each for index 1 and index 2.

### Bioinformatic analysis

The raw sequencing data were processed using the PETRI-seq pipeline (https://tavazoielab.c2b2.columbia.edu/PETRI-seq/). The top 50,000 barcoded cells with the highest UMI counts from each biological replicate were mapped to the *P. gingivalis* W83 reference genome (NCBI RefSeq GCF_002892595.1) [6]. To ensure transcript quality, we retained only cells with ≥15 UMIs mapped to protein-coding genes. This threshold is consistent with the criteria used in previous bacterial scRNA-seq studies employing PETRI-seq [4,7], where a similar minimum threshold was used during data preprocessing. This conservative cutoff helps to exclude low-quality or ambient RNA-derived barcodes while preserving informative biological variability.

To evaluate transcriptional heterogeneity and minimize technical artifacts, we performed three independent biological replicates and applied Canonical Correlation Analysis (CCA) using the Seurat v5.0.2 ‘IntegrateLayers’ function to integrate the replicates and correct for batch effects. This approach follows established practices in bacterial single-cell transcriptomics, where even single-batch designs have been shown sufficient to resolve meaningful transcriptional subpopulations [4,7]. UMAP (Uniform Manifold Approximation and Projection) visualization confirmed a high degree of intermixing among cells from different replicates, indicating successful batch correction and clustering robustness.

For downstream analyses, single-cell expression profiles were processed using the Seurat package (v5.0.2) in R (v4.4.1). Each replicate dataset was normalized and integrated *via* CCA. Cell clusters were identified using a graph-based clustering approach with resolution set to 0.35. For sub-clustering, cells from Clusters 0 and 1 were extracted and reclustered at a resolution of 0.5, resulting in four sub-clusters. Marker genes for each cluster and sub-cluster were identified using the ‘FindMarkers’ function in Seurat with the parameter min.pct = 0.1, selecting genes with an adjusted p-value <0.05. Functional annotation of marker genes was performed using eggNOG-mapper (http://eggnog-mapper.embl.de/).

## Results

### scRNA-seq workflow

We performed scRNA-seq using the split-pool barcoding protocol [4,5] optimized for *P. gingivalis*. As illustrated in Figure 1, cells were cultured to an OD_600_ of 0.35, fixed with 4% formaldehyde, and permeabilized in lysozyme-containing buffer. Fixed cells were distributed across 96 wells for split-pool barcoding, where they underwent three rounds of barcoding to tag transcripts at the single-cell level. Following RT and ligation steps, cells were lysed, and cDNA libraries were prepared. This workflow was applied to three independently prepared cultures to ensure reproducibility and minimize batch-specific artifacts.

### Quality control of the scRNA-seq data

**Figure 2.**
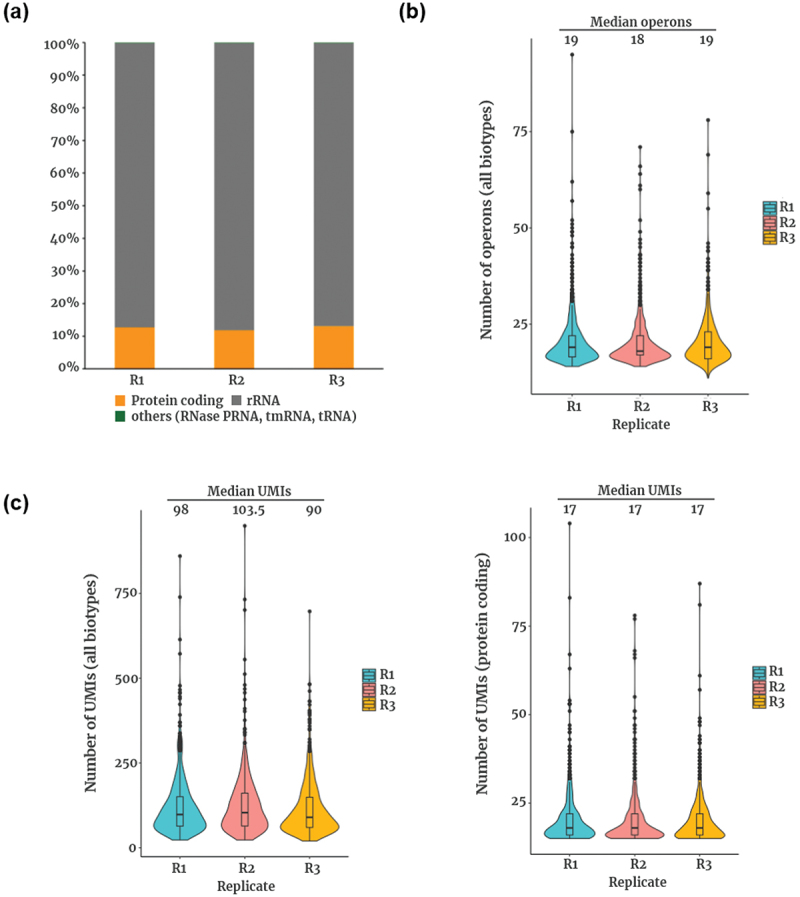
Overview of the expression profiles of *P. gingivalis* at the single-cell level. (a) composition of mapped UMIs across replicates. Mapped reads include five operon biotypes: protein-coding, rRNA, RNase PRNA, tmRNA, and tRNA. (b) Number of detected operons per cell across all biotypes. (c) Number of UMIs detected per cell in each replicate. Left: all biotype operons. Right: only protein-coding operons.

To validate data quality, we analyzed UMI composition, the number of detected operons, and transcript capture efficiency across all replicates. The UMI composition was consistent, with the majority of reads mapping to ribosomal RNA (rRNA), followed by protein-coding genes and other RNA species, including RNase P RNA, transfer-messenger RNA (tmRNA), and transfer RNA (tRNA) (Figure 2a). This distribution aligns with previous bacterial scRNA-seq studies, which have highlighted the challenge of high rRNA abundance in prokaryotic transcriptomes [4,5,7].

**Figure 3.**
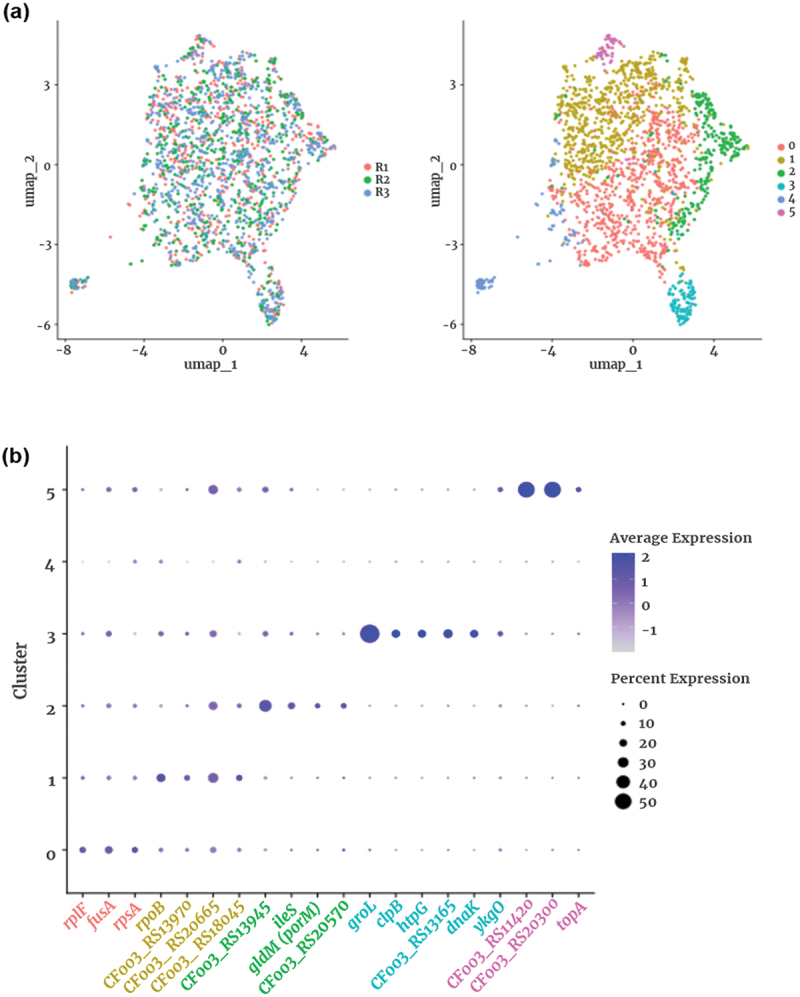
Single-cell transcriptome profiles reveal the heterogeneity of *P. gingivalis* cultures. (a) uniform manifold approximation and projection (UMAP) visualization of *P. gingivalis* single-cell transcriptomes. The left panel shows cells grouped by replicate, confirming minimal batch effects. The right panel displays six transcriptionally distinct clusters (0–5), with each dot representing a single *P. gingivalis* cell. Cluster sizes: cluster 0 (724 cells, 37.3%), cluster 1 (687 cells, 35.4%), cluster 2 (263 cells, 13.5%), cluster 3 (108 cells, 5.6%), cluster 4 (107 cells, 5.5%), cluster 5 (53 cells, 2.7%). (b) Dot plot showing the expression of the top 20 differentially expressed genes across the identified clusters. The size of each dot represents the percentage of cells expressing the gene within a given cluster, while color intensity indicates the average expression level.

The number of operons detected per cell was comparable across replicates, with a median of 18–19 operons per cell when considering all RNA biotypes (Figure 2b). The median UMI count per cell also remained stable across replicates, supporting the robustness and reproducibility of our dataset (Figure 2c). Furthermore, uniform transcriptomic patterns were observed across biological replicates, as demonstrated by the intermixing of replicate-derived cells in the UMAP projection (see Figure 3a, left panel), indicating minimal batch effects. When focusing exclusively on protein-coding operons, the number of detected operons per cell was lower, consistent with previous bacterial scRNA-seq studies, which have reported challenges in capturing messenger RNA (mRNA) due to its lower abundance compared to rRNA [7].

Cluster 4 is not included in this panel because no genes met the statistical threshold for unique marker selection (adjusted *p*<0.05).

Despite these technical challenges, our dataset demonstrates robust transcript capture, with UMI counts per cell comparable to or exceeding those reported in earlier bacterial scRNA-seq studies using split-pool barcoding [4,5]. These results confirm that our scRNA-seq dataset for *P. gingivalis* is of high quality and suitable for downstream analyses, including differential gene expression and subpopulation identification.

### Main clustering reveals six distinct transcriptional subpopulations

Following quality control filtering, we performed clustering analysis on a total of 1,942 high-quality *P. gingivalis* cells. The UMAP visualization (Figure 3a, left) demonstrated substantial intermixing of cells from the three replicates, indicating high reproducibility and minimal batch effects. Unsupervised clustering identified six transcriptionally distinct subpopulations (Clusters 0–5) (Figure 3a, right). Among these, Cluster 0 (724 cells, 37.3%) and Cluster 1 (687 cells, 35.4%) comprised the majority of the population, whereas Cluster 2 (263 cells, 13.5%), Cluster 3 (108 cells, 5.6%), Cluster 4 (107 cells, 5.5%), and Cluster 5 (53 cells, 2.7%) represented progressively smaller subpopulations. A summary of the top marker genes for each cluster is provided in Table S2.

To characterize the biological differences among these clusters, we performed differentially expressed gene (DEG) analysis (Figure 3b). Cluster 0 was enriched for ribosomal protein genes such as *rplF*, *fusA*, and *rpsA*, suggesting a metabolically active population primarily engaged in protein synthesis. Cluster 1 exhibited upregulation of *rpoB*, encoding the β-subunit of RNA polymerase, consistent with increased transcriptional activity. This cluster also expressed genes related to metabolism and transport, including *CF003_RS13970* (4-hydroxyphenylacetate 3-hydroxylase family protein) and *CF003_RS18045*, a TonB-dependent receptor from the *iht/tlr* iron acquisition locus. Although *CF003_RS18045* is not the canonical heme receptor HmuR (encoded by *CF003_RS18805*, see Sub-cluster C), its up-regulation suggests that a fraction of Cluster 1 cells is geared toward alternative iron-uptake pathways under iron-limited conditions [8–11]. The co-expression of transcriptional machinery and transport-related genes suggests that Cluster 1 may represent a functionally versatile or transitional subpopulation that is shifting between metabolic states, in line with asynchronous expression patterns observed in previous bacterial scRNA-seq studies [4,5,7].

Cluster 2 displayed distinct expression of *CF003_RS13945* (aldehyde dehydrogenase family protein), *ileS* (isoleucine – tRNA ligase), *gldM* (also known as *porM*, a component of the type IX secretion system [T9SS]), and *CF003_RS20570* (peptide Major Facilitator Superfamily [MFS] transporter). These genes suggest functional roles in amino acid metabolism and secretion of virulence factors. In particular, *gldM* (*porM*), a key component of the Type IX Secretion System, is essential for exporting surface-associated proteins, including proteases and adhesins that mediate host colonization and tissue invasion. Cluster 3 was characterized by elevated expression of stress response genes (*groL*, *clpB*, *htpG*, *dnaK*), which encode molecular chaperones that support protein folding and survival under environmental stress. Notably, the Clp system has been linked to biofilm formation and intracellular persistence in *P. gingivalis* [12]. The smallest subpopulation, Cluster 5, was distinguished by genes associated with DNA topology and proteolysis, including *topA* (Type I DNA topoisomerase) and *CF003_RS20300* (zinc-dependent metalloprotease), suggesting a role in DNA maintenance and protein turnover (Figure S1, Table S2).

**Figure 4.**
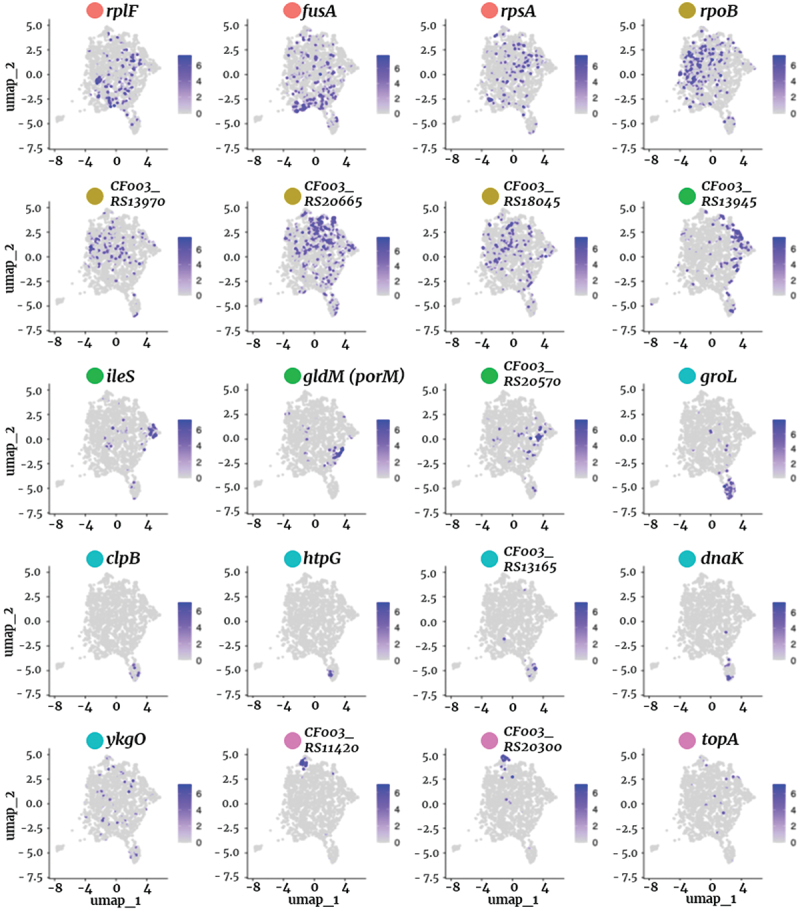
Spatial distribution of cluster-specific marker genes in *P. gingivalis*. Feature plots showing the expression patterns of 20 cluster-specific marker genes on the UMAP projection. Each panel represents a differentially expressed gene, with color intensity indicating the expression level in individual cells. These visualizations confirm the enrichment of specific genes within distinct subpopulations, further supporting the transcriptional heterogeneity of *P. gingivalis*.

To further validate these cluster-specific transcriptional profiles, we visualized key marker genes using feature plots (Figure 4). These plots confirmed distinct spatial expression patterns for representative genes, supporting the presence of functionally specialized subpopulations. The observed transcriptional heterogeneity in *P. gingivalis* reveals a spectrum of physiological states that may contribute to its adaptability and persistence in the host environment.

Cluster 4 is not included in this panel because no genes met the statistical threshold for unique marker selection (adjusted *p*<0.05).

### Sub-clustering reveals finer transcriptional heterogeneity within major clusters

**Figure 5.**
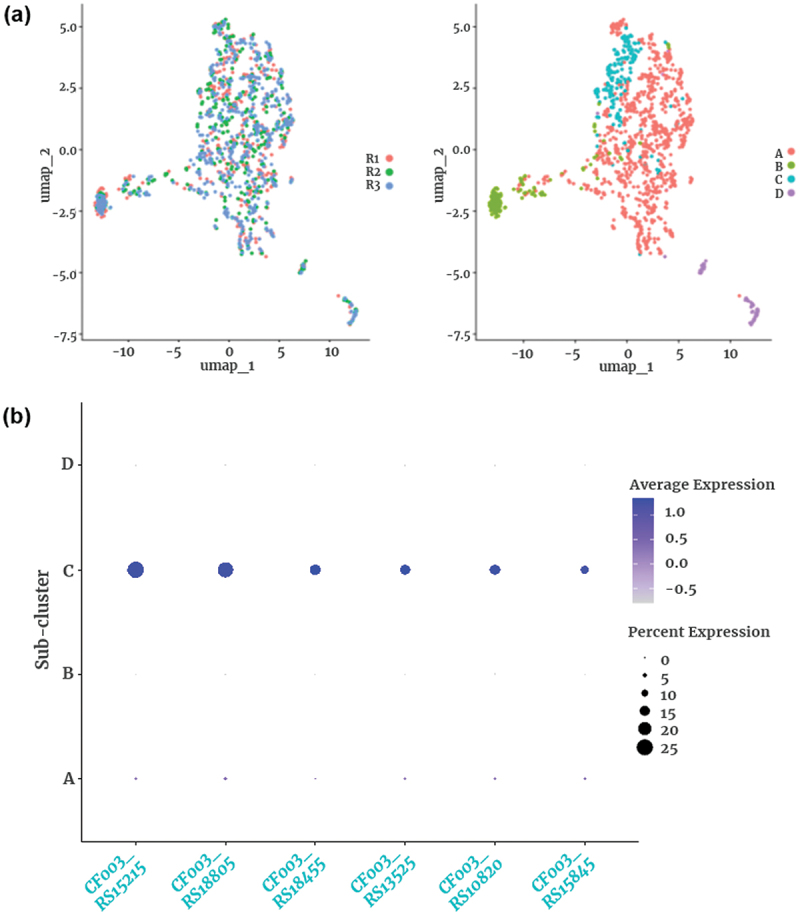
Sub-clustering of major clusters (0 and 1). (a) UMAP projection of sub-clusters within major clusters 0 and 1. The left panel shows cells grouped by replicate, while the right panel displays the four identified sub-clusters. Each dot represents a single *P. gingivalis* cell. Sub-cluster sizes: sub-cluster a (956 cells, 67.8%), sub-cluster B (199 cells, 14.1%), sub-cluster C (183 cells, 13.0%), sub-cluster D (73 cells, 5.2%). (b) Dot plot showing the expression of six marker genes identified in sub-cluster C.

To investigate additional transcriptional diversity within the dominant populations, we performed sub-clustering on Clusters 0 and 1, which together comprised 72.7% of the total cell population (1,411 out of 1,942 cells). This analysis resolved four distinct subpopulations: Sub-cluster A (956 cells, 67.8%), Sub-cluster B (199 cells, 14.1%), Sub-cluster C (183 cells, 13.0%), and Sub-cluster D (73 cells, 5.2%) (Figure 5a, right).

Gene expression analysis revealed that Sub-cluster C was notably enriched for genes involved in nutrient acquisition and stress adaptation (Figure 5b). For example, *CF003_RS18805* (*hmuR*) encoding the canonical TonB-dependent heme receptor, was upregulated, suggesting enhanced capacity for heme uptake – a critical determinant of *P. gingivalis* virulence [10]. In parallel, *CF003_RS18455*, encoding a heavy-metal P-type ATPase, and *CF003_RS10820*, encoding the ClpC ATP-binding subunit, were elevated, suggesting roles in metal homeostasis and proteostasis, respectively [12]. These expression patterns are further illustrated by violin plots (Figure S2) and feature plots on the UMAP projection (Figure S3).

Functional annotation of Sub-cluster C marker genes using eggNOG mapper revealed coordinated expression profiles in pathways related to metabolic flexibility, stress response, and virulence-associated resource acquisition. Specifically, *fhs* (formate – tetrahydrofolate ligase) participates in one-carbon metabolism and folate biosynthesis (Kyoto Encyclopedia of Genes and Genomes [KEGG]: ko00670, ko00720), supporting adaptive metabolic reprogramming. *clpC*, a Clp protease subunit (KEGG: ko01100), contributes to protein quality control under stress. *actP*, a heavy metal-translocating P-type ATPase (KEGG: ko01524, ko04016), may support detoxification under metal stress. These findings are detailed in Supplementary Table S4.

Together, these results highlight that *P. gingivalis*, even within its major transcriptional clusters, harbors subpopulation-level heterogeneity with distinct functional programs. Such diversity may underpin its capacity for environmental resilience, niche adaptation, and virulence within host environments.

## Discussion

scRNA-seq has transformed our understanding of cellular heterogeneity in eukaryotic systems, yet its application to prokaryotes remains in its early stages. This study represents the first application of scRNA-seq to *P. gingivalis*, revealing previously unrecognized transcriptional heterogeneity within an *in vitro*-grown population. Rather than existing as a transcriptionally uniform community, *P. gingivalis* exhibited distinct subpopulations with differential expression of genes related to protein synthesis, stress adaptation, metabolic activity, and membrane transport, suggesting functional diversification among individual cells. These findings align with previous reports that bacterial populations often contain rare but functionally important subpopulations, which can be challenging to detect using bulk transcriptomic analyses [4,5]. Our results reinforce the utility of scRNA-seq using split-pool barcoding for identifying these subpopulations, similar to previous studies where this approach detected rare bacterial subsets as small as 0.14–0.4% [4,5].

Bacterial heterogeneity is increasingly recognized as a key factor influencing microbial survival and pathogenicity [13–17]. In our study, metabolically active subpopulations were enriched for ribosomal protein genes, indicating that certain cells prioritize growth and proliferation, while others upregulated molecular chaperones and proteases, potentially enhancing stress tolerance. A particularly intriguing finding is the differential expression of genes involved in iron acquisition, protein degradation, and membrane transport within a specific subpopulation. *P. gingivalis* relies heavily on heme uptake for survival, and the upregulation of TonB-dependent receptors suggests that some cells may be specialized for iron acquisition [10]. Functional annotation of marker genes in this subpopulation also revealed coordinated expression profiles in genes associated with one-carbon metabolism, heavy-metal transport, and proteostasis, suggesting an adaptation to nutrient-limited and stress-inducing environments (Supplementary Table S4). This could provide a survival advantage in iron-limited environments, such as within host tissues or biofilms. If such functional differentiation enables adaptation to environmental pressures, it may contribute to *P. gingivalis* persistence in periodontal infections and its interactions with other members of the oral microbiome.

This transcriptional heterogeneity has significant implications for periodontal disease pathogenesis. Rather than acting as a single, homogeneous pathogen, *P. gingivalis* may exhibit phenotypic diversification that allows subpopulations to evade immune responses, persist within biofilms, and interact with other oral microbiota. These findings suggest a refinement of the conventional view of *P. gingivalis*, highlighting its potential to act through functionally specialized subpopulations within a broader pathogenic network. Furthermore, this heterogeneity may explain why *P. gingivalis* is difficult to eradicate using conventional antimicrobial therapies. If distinct subpopulations specialize in growth, persistence, or stress resistance, treating *P. gingivalis* as a single homogeneous entity may be insufficient. While our study does not directly assess antibiotic tolerance, single-cell analyses in other bacteria have identified subpopulations with reduced susceptibility to antibiotics due to metabolic dormancy or stress-adaptive responses [18,19]. Future studies should explore whether similar mechanisms exist in *P. gingivalis*, particularly in biofilm-associated infections.

While much research on the oral microbiome has focused on cataloging microbial diversity, relatively little attention has been given to the selective pressures and molecular mechanisms shaping this diversity [3]. Our findings highlight the need for further investigation into how genetic and transcriptional alterations within bacterial subsets influence their fitness, growth, and interactions with the broader microbial community. Understanding these processes may provide insights into whether selectively targeting certain bacterial subpopulations could shift the oral microbiome from dysbiosis to a more balanced state. In particular, future studies using scRNA-seq in *in vivo* models, biofilm settings, or co-culture with oral microbial consortia may provide critical insight into how host-mimicking environments and microbial interactions influence *P. gingivalis* subpopulation dynamics. Additionally, capturing and validating these changes at the single-cell level will be essential for developing precision-targeted therapeutic strategies.

One methodical limitation of our study is the high abundance of rRNA observed in the sequencing data (Figure 2a), which is a common and well-documented challenge in bacterial scRNA-seq [4,5]. We did not apply rRNA depletion to avoid potential biases and sample loss, which are critical concerns given the low RNA content of individual bacterial cells. Instead, we increased the read sequencing depth to improve mRNA capture. Despite the rRNA background, the clear transcriptional clusters and consistent marker gene expression imply that meaningful biological heterogeneity was successfully identified. Future studies utilizing optimized rRNA depletion protocols would give us more biological insights to understand the *P. gingivalis* populations and its heterogeneity.

A key limitation of our study is that all bacterial samples were cultured under standard laboratory conditions. While this experimental design minimized external variability and allowed for the detection of intrinsic transcriptional heterogeneity, it remains unclear how these subpopulations behave *in vivo*. Host immune pressures, interspecies interactions, and environmental stressors likely influence *P. gingivalis* heterogeneity, potentially leading to dynamic shifts in transcriptional states. The spatial structure of biofilms and host-derived signals are thought to influence bacterial behavior and gene expression, potentially inducing heterogeneity even within clonal populations [20,21]. Investigating whether such mechanisms operate in *P. gingivalis* will be important for understanding its *in situ* physiology.

In conclusion, our findings challenge the traditional view of *P. gingivalis* as a genetically homogeneous pathogen by demonstrating its existence as a collection of transcriptionally distinct subpopulations. This heterogeneity likely contributes to its resilience, virulence, and persistence within the host environment. However, our study is limited by its reliance on *in vitro* cultures and technical constraints associated with bacterial scRNA-seq, such as high rRNA content and low mRNA yield. Moving forward, it will be critical to investigate how these subpopulations dynamically shift in response to host immune pressures, antimicrobial treatments, and disease progression. By leveraging single-cell technologies, future research may uncover novel bacterial regulatory mechanisms, ultimately informing more precise therapeutic strategies that target bacterial heterogeneity.

## Supplementary Material

DocumentS1_revclean.pdf

## Data Availability

The raw RNA sequencing data generated in this study have been deposited in the NCBI Gene Expression Omnibus (GEO) database under the accession number GSE290203. For the review purpose, go to https://www.ncbi.nlm.nih.gov/geo/query/acc.cgi?acc=GSE290203. Enter token qhqjkgyuddurlil into the box.
