# Supplementary material for: Single-cell RNA sequencing using split-pool barcoding reveals transcriptional heterogeneity in Porphyromonas gingivalis with implications for periodontal pathogenesis
Source: J Oral Microbiol. 2025 Jul 31;17(1):2540827. doi: 10.1080/20002297.2025.2540827 (PMC12315123; doi:10.1080/20002297.2025.2540827)
Supplement: DocumentS1_revclean.pdf [file ZJOM_A_2540827_SM1368.pdf]

Single-cell RNA sequencing using split-pool barcoding reveals transcriptional heterogeneity in *Porphyromonas gingivalis* with implications for periodontal pathogenesis

Eun-Young Jang, Seok Bin Yang, Jeewan Chun, Kyu Hwan Kwack, Sang-Wook Kang, Jae-Hyung Lee, Ji-Hoi Moon

Table S1. Oligonucleotides used in this study. Detailed information about the barcode design can be found at https://tavazoielab.c2b2.columbia.edu/PETRI-seq.

Round 2 Linker Oligo (L2)	STCTGGCGTAGGAGGW
Round 3 Linker Oligo (L3)	GCGAAGCCAAGGACCW
Round 2 Blocking 1 (B2-1)	GCCAGASACGTTAGGCAGGACCTAACGT
Round 3 Blocking 1 (B3-1)	GCTTCGCTGCAATCGGACCTCGATTGCA
Round 2 Blocking 2 (B2-2)	WCCTCCTACGCCAGAS
Round 3 Blocking 2 (B3-2)	WGGTCCTTGGCTTCGC

Barcode 1 RT	Barcode 2 Ligation	Barcode 3 Ligation
1 /5Phos/GCCAGACAGAGAANNNNNNN	1 /5Phos/GCTTCGCGACCTTACCTCCTAC	1 AGAATACACGACGCTCTTCCGATCTNNNNNNNGTGTGAAGGTCCTTG
2 /5Phos/GCCAGACCAGGAANNNNNNN	2 /5Phos/GCTTCGCAGAGTGACCTCCTAC	2 AGAATACACGACGCTCTTCCGATCTNNNNNNNTTGGTGAGGTCCTTG
3 /5Phos/GCCAGACTCCGAANNNNNNN	3 /5Phos/GCTTCGCTGACTCACCTCCTAC	3 AGAATACACGACGCTCTTCCGATCTNNNNNNNGTCACAAGGTCCTTG
4 /5Phos/GCCAGACGTTGAANNNNNNN	4 /5Phos/GCTTCGCGCCTGTACCTCCTAC	4 AGAATACACGACGCTCTTCCGATCTNNNNNNNGCGATAAGGTCCTTG
5 /5Phos/GCCAGACCTACAANNNNNNN	5 /5Phos/GCTTCGCGTAGTCACCTCCTAC	5 AGAATACACGACGCTCTTCCGATCTNNNNNNNTACAGCAGGTCCTTG
6 /5Phos/GCCAGACTGTCAANNNNNNN	6 /5Phos/GCTTCGCTGGCGAACCTCCTAC	6 AGAATACACGACGCTCTTCCGATCTNNNNNNNTAGGCAAGGTCCTTG
7 /5Phos/GCCAGACGCATAANNNNNNN	7 /5Phos/GCTTCGCGCTGCTACCTCCTAC	7 AGAATACACGACGCTCTTCCGATCTNNNNNNNAACGTGAGGTCCTTG
8 /5Phos/GCCAGACAAGAGANNNNNNN	8 /5Phos/GCTTCGCACCAGAACCTCCTAC	8 AGAATACACGACGCTCTTCCGATCTNNNNNNNGGTTCAAGGTCCTTG
9 /5Phos/GCCAGACTGCAGANNNNNNN	9 /5Phos/GCTTCGCGGTTCAACCTCCTAC	9 AGAATACACGACGCTCTTCCGATCTNNNNNNNGAGAAGAGGTCCTTG
10 /5Phos/GCCAGACGAACGANNNNNNN	10 /5Phos/GCTTCGCGGACAGACCTCCTAC	10 AGAATACACGACGCTCTTCCGATCTNNNNNNNCGCGATAGGTCCTTG
11 /5Phos/GCCAGACTTGTGANNNNNNN	11 /5Phos/GCTTCGCCATGGTACCTCCTAC	11 AGAATACACGACGCTCTTCCGATCTNNNNNNNAAGTCGAGGTCCTTG
12 /5Phos/GCCAGACCGTTGANNNNNNN	12 /5Phos/GCTTCGCACGGAGACCTCCTAC	12 AGAATACACGACGCTCTTCCGATCTNNNNNNNCCAGCTAGGTCCTTG
13 /5Phos/GCCAGACGGAACANNNNNNN	13 /5Phos/GCTTCGCCTAACGACCTCCTAC	13 AGAATACACGACGCTCTTCCGATCTNNNNNNNCCGAATAGGTCCTTG
14 /5Phos/GCCAGACCTCACANNNNNNN	14 /5Phos/GCTTCGCTCCGTAACCTCCTAC	14 AGAATACACGACGCTCTTCCGATCTNNNNNNNTCAGGAAGGTCCTTG
15 /5Phos/GCCAGACACTACANNNNNNN	15 /5Phos/GCTTCGCCACTCAACCTCCTAC	15 AGAATACACGACGCTCTTCCGATCTNNNNNNNGACGGAAGGTCCTTG
16 /5Phos/GCCAGACTAAGCANNNNNNN	16 /5Phos/GCTTCGCCAGAGAACCTCCTAC	16 AGAATACACGACGCTCTTCCGATCTNNNNNNNCACACGAGGTCCTTG
17 /5Phos/GCCAGACGAGTCANNNNNNN	17 /5Phos/GCTTCGCTGCACTACCTCCTAC	17 AGAATACACGACGCTCTTCCGATCTNNNNNNNATGAGGAGGTCCTTG
18 /5Phos/GCCAGACGCGATANNNNNNN	18 /5Phos/GCTTCGCATGAGGACCTCCTAC	18 AGAATACACGACGCTCTTCCGATCTNNNNNNNCTGCAGAGGTCCTTG
19 /5Phos/GCCAGACATGGTANNNNNNN	19 /5Phos/GCTTCGCACGCGTACCTCCTAC	19 AGAATACACGACGCTCTTCCGATCTNNNNNNNACCAGAAGGTCCTTG
20 /5Phos/GCCAGACACACTANNNNNNN	20 /5Phos/GCTTCGCATCGCCACCTCCTAC	20 AGAATACACGACGCTCTTCCGATCTNNNNNNNATAGCGAGGTCCTTG
21 /5Phos/GCCAGACTACCTANNNNNNN	21 /5Phos/GCTTCGCGTCGAGACCTCCTAC	21 AGAATACACGACGCTCTTCCGATCTNNNNNNNGTGGCTAGGTCCTTG
22 /5Phos/GCCAGACTTGAAGNNNNNNN	22 /5Phos/GCTTCGCTTGACCACCTCCTAC	22 AGAATACACGACGCTCTTCCGATCTNNNNNNNGGACAGAGGTCCTTG
23 /5Phos/GCCAGACGACAAGNNNNNNN	23 /5Phos/GCTTCGCTCAAGGACCTCCTAC	23 AGAATACACGACGCTCTTCCGATCTNNNNNNNCGTAGCAGGTCCTTG
24 /5Phos/GCCAGACAGTAAGNNNNNNN	24 /5Phos/GCTTCGCAGTCCTACCTCCTAC	24 AGAATACACGACGCTCTTCCGATCTNNNNNNNGAGCGTAGGTCCTTG
25 /5Phos/GCCAGACACGTAGNNNNNNN	25 /5Phos/GCTTCGCATCCTGACCTCCTAC	25 AGAATACACGACGCTCTTCCGATCTNNNNNNNCGTCCTAGGTCCTTG
26 /5Phos/GCCAGACCTCTAGNNNNNNN	26 /5Phos/GCTTCGCAGGACAACCTCCTAC	26 AGAATACACGACGCTCTTCCGATCTNNNNNNNCCTACAAGGTCCTTG
27 /5Phos/GCCAGACACAAGGNNNNNNN	27 /5Phos/GCTTCGCCACGTCACCTCCTAC	27 AGAATACACGACGCTCTTCCGATCTNNNNNNNTCCATGAGGTCCTTG
28 /5Phos/GCCAGACAATCGGNNNNNNN	28 /5Phos/GCTTCGCGCTTAGACCTCCTAC	28 AGAATACACGACGCTCTTCCGATCTNNNNNNNTGGAACAGGTCCTTG
29 /5Phos/GCCAGACCAATGGNNNNNNN	29 /5Phos/GCTTCGCGCCAACACCTCCTAC	29 AGAATACACGACGCTCTTCCGATCTNNNNNNNACAACCAGGTCCTTG
30 /5Phos/GCCAGACTATACGNNNNNNN	30 /5Phos/GCTTCGCTTCGGTACCTCCTAC	30 AGAATACACGACGCTCTTCCGATCTNNNNNNNCCATTCAGGTCCTTG
31 /5Phos/GCCAGACATAGCGNNNNNNN	31 /5Phos/GCTTCGCGAGAAGACCTCCTAC	31 AGAATACACGACGCTCTTCCGATCTNNNNNNNCAGAGAAGGTCCTTG
32 /5Phos/GCCAGACTGATCGNNNNNNN	32 /5Phos/GCTTCGCCAGTTGACCTCCTAC	32 AGAATACACGACGCTCTTCCGATCTNNNNNNNTGGTCTAGGTCCTTG
33 /5Phos/GCCAGACAACTCGNNNNNNN	33 /5Phos/GCTTCGCTCAGACACCTCCTAC	33 AGAATACACGACGCTCTTCCGATCTNNNNNNNAGAGACAGGTCCTTG
34 /5Phos/GCCAGACCGAATGNNNNNNN	34 /5Phos/GCTTCGCCTTGACACCTCCTAC	34 AGAATACACGACGCTCTTCCGATCTNNNNNNNTCACACAGGTCCTTG
35 /5Phos/GCCAGACTCCATGNNNNNNN	35 /5Phos/GCTTCGCCCGGTTACCTCCTAC	35 AGAATACACGACGCTCTTCCGATCTNNNNNNNCACTACAGGTCCTTG
36 /5Phos/GCCAGACGTTATGNNNNNNN	36 /5Phos/GCTTCGCGATGTGACCTCCTAC	36 AGAATACACGACGCTCTTCCGATCTNNNNNNNGGTGTTAGGTCCTTG
37 /5Phos/GCCAGACCATGTGNNNNNNN	37 /5Phos/GCTTCGCCTGGCAACCTCCTAC	37 AGAATACACGACGCTCTTCCGATCTNNNNNNNGGCTTGAGGTCCTTG
38 /5Phos/GCCAGACTTACTGNNNNNNN	38 /5Phos/GCTTCGCGAACCAACCTCCTAC	38 AGAATACACGACGCTCTTCCGATCTNNNNNNNGAATGGAGGTCCTTG
39 /5Phos/GCCAGACTAGTTGNNNNNNN	39 /5Phos/GCTTCGCCAAGAGACCTCCTAC	39 AGAATACACGACGCTCTTCCGATCTNNNNNNNGTAGGCAGGTCCTTG
40 /5Phos/GCCAGACTCGCATNNNNNNN	40 /5Phos/GCTTCGCGCTCGAACCTCCTAC	40 AGAATACACGACGCTCTTCCGATCTNNNNNNNTCTCCGAGGTCCTTG
41 /5Phos/GCCAGACCGCAATNNNNNNN	41 /5Phos/GCTTCGCCGCATAACCTCCTAC	41 AGAATACACGACGCTCTTCCGATCTNNNNNNNAGCTCCAGGTCCTTG
42 /5Phos/GCCAGACGCTAATNNNNNNN	42 /5Phos/GCTTCGCCGGAACACCTCCTAC	42 AGAATACACGACGCTCTTCCGATCTNNNNNNNCCTGACAGGTCCTTG
43 /5Phos/GCCAGACATCCATNNNNNNN	43 /5Phos/GCTTCGCCCTTGCACCTCCTAC	43 AGAATACACGACGCTCTTCCGATCTNNNNNNNGGAAGAAGGTCCTTG
44 /5Phos/GCCAGACCTGAGTNNNNNNN	44 /5Phos/GCTTCGCCCACATACCTCCTAC	44 AGAATACACGACGCTCTTCCGATCTNNNNNNNATCCGCAGGTCCTTG
45 /5Phos/GCCAGACTTCGGTNNNNNNN	45 /5Phos/GCTTCGCCAACGCACCTCCTAC	45 AGAATACACGACGCTCTTCCGATCTNNNNNNNGCCTGTAGGTCCTTG
46 /5Phos/GCCAGACGATGGTNNNNNNN	46 /5Phos/GCTTCGCCCAATCACCTCCTAC	46 AGAATACACGACGCTCTTCCGATCTNNNNNNNTGGCGAAGGTCCTTG
47 /5Phos/GCCAGACAGACGTNNNNNNN	47 /5Phos/GCTTCGCCGTAGGACCTCCTAC	47 AGAATACACGACGCTCTTCCGATCTNNNNNNNGCAGTGAGGTCCTTG
48 /5Phos/GCCAGACCTTCTTNNNNNNN	48 /5Phos/GCTTCGCTGTGAGACCTCCTAC	48 AGAATACACGACGCTCTTCCGATCTNNNNNNNGAACCAAGGTCCTTG
49 /5Phos/GCCAGAGACGGAANNNNNNN	49 /5Phos/GCTTCGCACTGGTTCCTCCTAC	49 AGAATACACGACGCTCTTCCGATCTNNNNNNNTAGGAGTGGTCCTTG
50 /5Phos/GCCAGAGCTCGAANNNNNNN	50 /5Phos/GCTTCGCAAGCGATCCTCCTAC	50 AGAATACACGACGCTCTTCCGATCTNNNNNNNCTGGACTGGTCCTTG
51 /5Phos/GCCAGAGTCACAANNNNNNN	51 /5Phos/GCTTCGCGGCCATTCCTCCTAC	51 AGAATACACGACGCTCTTCCGATCTNNNNNNNGTTCGCTGGTCCTTG
52 /5Phos/GCCAGAGAACCAANNNNNNN	52 /5Phos/GCTTCGCAACGAGTCCTCCTAC	52 AGAATACACGACGCTCTTCCGATCTNNNNNNNTTGCCTTGGTCCTTG
53 /5Phos/GCCAGAGCGATAANNNNNNN	53 /5Phos/GCTTCGCACCATCTCCTCCTAC	53 AGAATACACGACGCTCTTCCGATCTNNNNNNNATCACCTGGTCCTTG
54 /5Phos/GCCAGAGGTGTAANNNNNNN	54 /5Phos/GCTTCGCCATTGGTCCTCCTAC	54 AGAATACACGACGCTCTTCCGATCTNNNNNNNAATGCCTGGTCCTTG
55 /5Phos/GCCAGAGAGAAGANNNNNNN	55 /5Phos/GCTTCGCGAGTCATCCTCCTAC	55 AGAATACACGACGCTCTTCCGATCTNNNNNNNGGAATGTGGTCCTTG
56 /5Phos/GCCAGAGTCGAGANNNNNNN	56 /5Phos/GCTTCGCCGCTAATCCTCCTAC	56 AGAATACACGACGCTCTTCCGATCTNNNNNNNGCGGATTGGTCCTTG
57 /5Phos/GCCAGAGGACAGANNNNNNN	57 /5Phos/GCTTCGCATCCACTCCTCCTAC	57 AGAATACACGACGCTCTTCCGATCTNNNNNNNCTGTGGTGGTCCTTG
58 /5Phos/GCCAGAGCTTAGANNNNNNN	58 /5Phos/GCTTCGCGATCAGTCCTCCTAC	58 AGAATACACGACGCTCTTCCGATCTNNNNNNNCGATCCTGGTCCTTG
59 /5Phos/GCCAGAGAATGGANNNNNNN	59 /5Phos/GCTTCGCAGAGACTCCTCCTAC	59 AGAATACACGACGCTCTTCCGATCTNNNNNNNTAAGGCTGGTCCTTG
60 /5Phos/GCCAGAGTTCCGANNNNNNN	60 /5Phos/GCTTCGCGCACAATCCTCCTAC	60 AGAATACACGACGCTCTTCCGATCTNNNNNNNGCCACTTGGTCCTTG
61 /5Phos/GCCAGAGCAGTGANNNNNNN	61 /5Phos/GCTTCGCGTGTAGTCCTCCTAC	61 AGAATACACGACGCTCTTCCGATCTNNNNNNNGGCCATTGGTCCTTG
62 /5Phos/GCCAGAGCCAACANNNNNNN	62 /5Phos/GCTTCGCAGCTCGTCCTCCTAC	62 AGAATACACGACGCTCTTCCGATCTNNNNNNNACGAGTTGGTCCTTG
63 /5Phos/GCCAGAGATGACANNNNNNN	63 /5Phos/GCTTCGCAGGCAGTCCTCCTAC	63 AGAATACACGACGCTCTTCCGATCTNNNNNNNCGAGGTTGGTCCTTG
64 /5Phos/GCCAGAGTGTACANNNNNNN	64 /5Phos/GCTTCGCCAAGCATCCTCCTAC	64 AGAATACACGACGCTCTTCCGATCTNNNNNNNTTCTGCTGGTCCTTG
65 /5Phos/GCCAGAGGTACCANNNNNNN	65 /5Phos/GCTTCGCTCCACATCCTCCTAC	65 AGAATACACGACGCTCTTCCGATCTNNNNNNNCAGGCTTGGTCCTTG
66 /5Phos/GCCAGAGTACTCANNNNNNN	66 /5Phos/GCTTCGCTAGAGGTCCTCCTAC	66 AGAATACACGACGCTCTTCCGATCTNNNNNNNTCCGGTTGGTCCTTG
67 /5Phos/GCCAGAGCGCATANNNNNNN	67 /5Phos/GCTTCGCGTCAGGTCCTCCTAC	67 AGAATACACGACGCTCTTCCGATCTNNNNNNNATGGCATGGTCCTTG
68 /5Phos/GCCAGAGTAGGTANNNNNNN	68 /5Phos/GCTTCGCCGAGGTTCCTCCTAC	68 AGAATACACGACGCTCTTCCGATCTNNNNNNNTGCGTGTGGTCCTTG
69 /5Phos/GCCAGAGCCTGTANNNNNNN	69 /5Phos/GCTTCGCAGTCGCTCCTCCTAC	69 AGAATACACGACGCTCTTCCGATCTNNNNNNNGCGTTGTGGTCCTTG
70 /5Phos/GCCAGAGCAACTANNNNNNN	70 /5Phos/GCTTCGCGAGGACTCCTCCTAC	70 AGAATACACGACGCTCTTCCGATCTNNNNNNNGGTGAATGGTCCTTG
71 /5Phos/GCCAGAGAGGCTANNNNNNN	71 /5Phos/GCTTCGCTGCAACTCCTCCTAC	71 AGAATACACGACGCTCTTCCGATCTNNNNNNNGAGAGCTGGTCCTTG
72 /5Phos/GCCAGAGACCTTANNNNNNN	72 /5Phos/GCTTCGCGTAACCTCCTCCTAC	72 AGAATACACGACGCTCTTCCGATCTNNNNNNNACTCTGTGGTCCTTG
73 /5Phos/GCCAGAGAAGAAGNNNNNNN	73 /5Phos/GCTTCGCATCGGATCCTCCTAC	73 AGAATACACGACGCTCTTCCGATCTNNNNNNNGATCCTTGGTCCTTG
74 /5Phos/GCCAGAGCCTAAGNNNNNNN	74 /5Phos/GCTTCGCTAACCGTCCTCCTAC	74 AGAATACACGACGCTCTTCCGATCTNNNNNNNAGCCGATGGTCCTTG
75 /5Phos/GCCAGAGTGAGAGNNNNNNN	75 /5Phos/GCTTCGCCCTGAATCCTCCTAC	75 AGAATACACGACGCTCTTCCGATCTNNNNNNNACCGACTGGTCCTTG
76 /5Phos/GCCAGAGATACAGNNNNNNN	76 /5Phos/GCTTCGCCACCTGTCCTCCTAC	76 AGAATACACGACGCTCTTCCGATCTNNNNNNNAACAGGTGGTCCTTG
77 /5Phos/GCCAGAGGATCAGNNNNNNN	77 /5Phos/GCTTCGCCCGTGATCCTCCTAC	77 AGAATACACGACGCTCTTCCGATCTNNNNNNNAGGATCTGGTCCTTG
78 /5Phos/GCCAGAGAGCTAGNNNNNNN	78 /5Phos/GCTTCGCGGTAGTTCCTCCTAC	78 AGAATACACGACGCTCTTCCGATCTNNNNNNNGCACGTTGGTCCTTG
79 /5Phos/GCCAGAGATCAGGNNNNNNN	79 /5Phos/GCTTCGCCGTATCTCCTCCTAC	79 AGAATACACGACGCTCTTCCGATCTNNNNNNNGAGCAATGGTCCTTG
80 /5Phos/GCCAGAGTAACGGNNNNNNN	80 /5Phos/GCTTCGCGTTGCATCCTCCTAC	80 AGAATACACGACGCTCTTCCGATCTNNNNNNNGGTACCTGGTCCTTG
81 /5Phos/GCCAGAGGTATGGNNNNNNN	81 /5Phos/GCTTCGCAAGACCTCCTCCTAC	81 AGAATACACGACGCTCTTCCGATCTNNNNNNNCTAGTGTGGTCCTTG
82 /5Phos/GCCAGAGACATCGNNNNNNN	82 /5Phos/GCTTCGCTGGTCTTCCTCCTAC	82 AGAATACACGACGCTCTTCCGATCTNNNNNNNTCAGCGTGGTCCTTG
83 /5Phos/GCCAGAGTTGTCGNNNNNNN	83 /5Phos/GCTTCGCTACCGTTCCTCCTAC	83 AGAATACACGACGCTCTTCCGATCTNNNNNNNTGGAGGTGGTCCTTG
84 /5Phos/GCCAGAGCATTCGNNNNNNN	84 /5Phos/GCTTCGCTCTGCCTCCTCCTAC	84 AGAATACACGACGCTCTTCCGATCTNNNNNNNCGCTAGTGGTCCTTG
85 /5Phos/GCCAGAGGCAATGNNNNNNN	85 /5Phos/GCTTCGCCCGAAGTCCTCCTAC	85 AGAATACACGACGCTCTTCCGATCTNNNNNNNCTGCTATGGTCCTTG
86 /5Phos/GCCAGAGTGGATGNNNNNNN	86 /5Phos/GCTTCGCCTCGCTTCCTCCTAC	86 AGAATACACGACGCTCTTCCGATCTNNNNNNNTCGACATGGTCCTTG
87 /5Phos/GCCAGAGCTAGTGNNNNNNN	87 /5Phos/GCTTCGCAGGTTCTCCTCCTAC	87 AGAATACACGACGCTCTTCCGATCTNNNNNNNAGGCAGTGGTCCTTG
88 /5Phos/GCCAGAGAACGTGNNNNNNN	88 /5Phos/GCTTCGCTCTCGGTCCTCCTAC	88 AGAATACACGACGCTCTTCCGATCTNNNNNNNACATGGTGGTCCTTG
89 /5Phos/GCCAGAGTCTCTGNNNNNNN	89 /5Phos/GCTTCGCGGCGTATCCTCCTAC	89 AGAATACACGACGCTCTTCCGATCTNNNNNNNACGTCCTGGTCCTTG
90 /5Phos/GCCAGAGACCAATNNNNNNN	90 /5Phos/GCTTCGCAGAAGGTCCTCCTAC	90 AGAATACACGACGCTCTTCCGATCTNNNNNNNCAGTTCTGGTCCTTG
91 /5Phos/GCCAGAGCATGATNNNNNNN	91 /5Phos/GCTTCGCACGGCATCCTCCTAC	91 AGAATACACGACGCTCTTCCGATCTNNNNNNNCAACGGTGGTCCTTG
92 /5Phos/GCCAGAGTCTGGTNNNNNNN	92 /5Phos/GCTTCGCCTGAGCTCCTCCTAC	92 AGAATACACGACGCTCTTCCGATCTNNNNNNNCGCACATGGTCCTTG
93 /5Phos/GCCAGAGCTACGTNNNNNNN	93 /5Phos/GCTTCGCATGGTGTCCTCCTAC	93 AGAATACACGACGCTCTTCCGATCTNNNNNNNGGTTGGTGGTCCTTG
94 /5Phos/GCCAGAGAAGCGTNNNNNNN	94 /5Phos/GCTTCGCCGATCCTCCTCCTAC	94 AGAATACACGACGCTCTTCCGATCTNNNNNNNCCAGAATGGTCCTTG
95 /5Phos/GCCAGAGTGCTGTNNNNNNN	95 /5Phos/GCTTCGCCCAACTTCCTCCTAC	95 AGAATACACGACGCTCTTCCGATCTNNNNNNNCTTCCGTGGTCCTTG
96 /5Phos/GCCAGAGTAGACTNNNNNNN	96 /5Phos/GCTTCGCGTGCTCTCCTCCTAC	96 AGAATACACGACGCTCTTCCGATCTNNNNNNNGCATACTGGTCCTTG

Table S2. Top marker genes of the six major clusters (0–5) in *P. gingivalis.* Note: Cluster 4 is not listed because no genes met the significance threshold for unique marker selection (adjusted $p < 0.05$).

cluster	gene	p-value	avg_log2FC	pct.1	pct.2	adj. p-value	product
0	*rplF*	8.69E-15	1.6932006	0.155	0.052	1.56E-11	50S ribosomal protein L6
0	*fusA*	2.04E-11	1.1494198	0.202	0.095	3.65E-08	elongation factor G
0	*rpsA*	1.32E-06	0.9800559	0.145	0.076	2.37E-03	30S ribosomal protein S1
1	*rpoB*	8.64E-21	1.7833057	0.227	0.08	1.55E-17	DNA-directed RNA polymerase subunit beta
1	*CF003_RS13970*	1.17E-11	1.6415864	0.137	0.05	2.09E-08	4-hydroxyphenylacetate 3-hydroxylase family protein
1	*CF003_RS20665*	2.47E-10	0.8763213	0.29	0.168	4.43E-07	TapA – T9SS cargo/type A sorting protein (part of a virulence operon)
1	*CF003_RS18045*	2.67E-06	1.0976676	0.147	0.08	4.78E-03	TonB-dependent receptor
2	*CF003_RS13945*	7.54E-56	2.9160597	0.354	0.052	1.35E-52	aldehyde dehydrogenase family protein
2	*ileS*	8.49E-32	3.3377399	0.175	0.021	1.52E-28	isoleucine--tRNA ligase
2	*gldM*	1.34E-19	3.3140947	0.11	0.014	2.40E-16	gliding motility protein GldM
2	*CF003_RS20570*	4.12E-15	2.4217274	0.129	0.027	7.38E-12	peptide MFS transporter
3	*groL*	3.99E-181	6.3823744	0.583	0.011	7.15E-178	chaperonin GroEL
3	*clpB*	6.70E-88	17.7560948	0.213	0	1.20E-84	ATP-dependent chaperone ClpB
3	*htpG*	4.05E-84	17.5138657	0.204	0	7.26E-81	molecular chaperone HtpG
3	*CF003_RS13165*	3.96E-82	6.7246458	0.241	0.003	7.09E-79	co-chaperone GroES
3	*dnaK*	2.94E-70	6.8623334	0.204	0.002	5.26E-67	molecular chaperone DnaK
3	*ykgO*	2.03E-05	1.689294	0.12	0.037	3.63E-02	type B 50S ribosomal protein L36
5	*CF003_RS11420*	3.61E-178	8.3840045	0.491	0.002	6.47E-175	aminoacyl-histidine dipeptidase
5	*CF003_RS20300*	2.45E-152	7.3372027	0.491	0.005	4.38E-149	zinc-dependent metalloprotease
5	*topA*	4.10E-12	4.1693895	0.113	0.009	7.35E-09	type I DNA topoisomerase

Table S3. Top marker genes of sub-cluster C within clusters 0 and 1

cluster	gene	p-value	avg_log2FC	pct.1	pct.2	adj. p-value	product
C	*CF003_RS15215*	5.40E-44	3.7903782	0.251	0.017	9.68E-41	formate--tetrahydrofolate ligase
C	*CF003_RS18805*	1.74E-35	3.761247	0.235	0.022	3.11E-32	TonB-dependent receptor
C	*CF003_RS18455*	7.28E-31	4.1070559	0.158	0.008	1.30E-27	heavy metal translocating P-type ATPase
C	*CF003_RS13525*	1.06E-24	3.9074795	0.148	0.011	1.89E-21	Do family serine endopeptidase
C	*CF003_RS10820*	1.23E-23	3.7759203	0.153	0.014	2.20E-20	ATP-dependent Clp protease ATP-binding subunit
C	*CF003_RS15845*	9.66E-16	3.0971057	0.115	0.013	1.73E-12	MMPL family transporter

Table S4. Functional annotations of sub-cluster C marker genes in *P. gingivalis*. This table summarizes the predicted functions of representative marker genes from sub-cluster C, including their orthologous group classifications (COG) and associated KEGG pathways.

gene	RefSeq ID	COG category	Preferred name	KEGG pathway	Description
CF003_RS15215	WP_004585540.1	F	fhs	ko00670,ko00720,ko01100, ko01120,ko01200	Belongs to the formate--tetrahydrofolate ligase family
CF003_RS18805	WP_005874477.1	H	-	-	Outer membrane protein beta-barrel family
CF003_RS18455	WP_005874448.1	P	actP	ko01524,ko04016	Copper-exporting ATPase
CF003_RS13525	WP_005874875.1	O	degQ	-	deoxyribonuclease HsdR
CF003_RS10820	WP_005873897.1	O	clpC	ko01100	Belongs to the ClpA ClpB family
CF003_RS15845	WP_010956241.1	S	-	-	Sterol-sensing domain of SREBP cleavage-activation

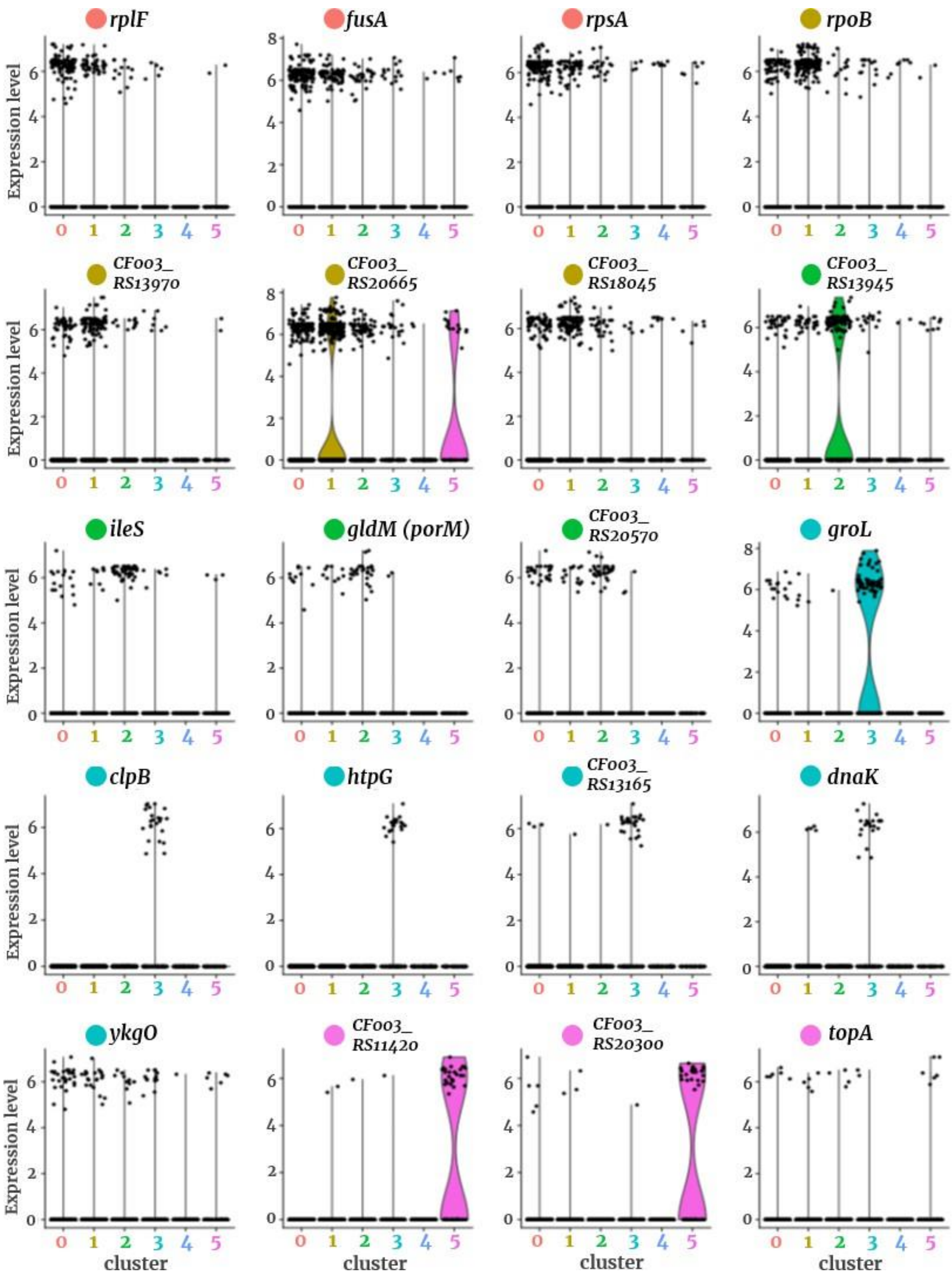

Figure S1. Cluster-specific marker genes identified in *P. gingivalis*. Violin plots depicting the expression distribution of 20 marker genes across clusters 0, 1, 2, 3, and 5. Each plot represents a gene specifically enriched in one or more clusters. **Note:** Cluster 4 is not included in this panel because no genes met the statistical threshold for unique marker selection (adjusted $p < 0.05$).

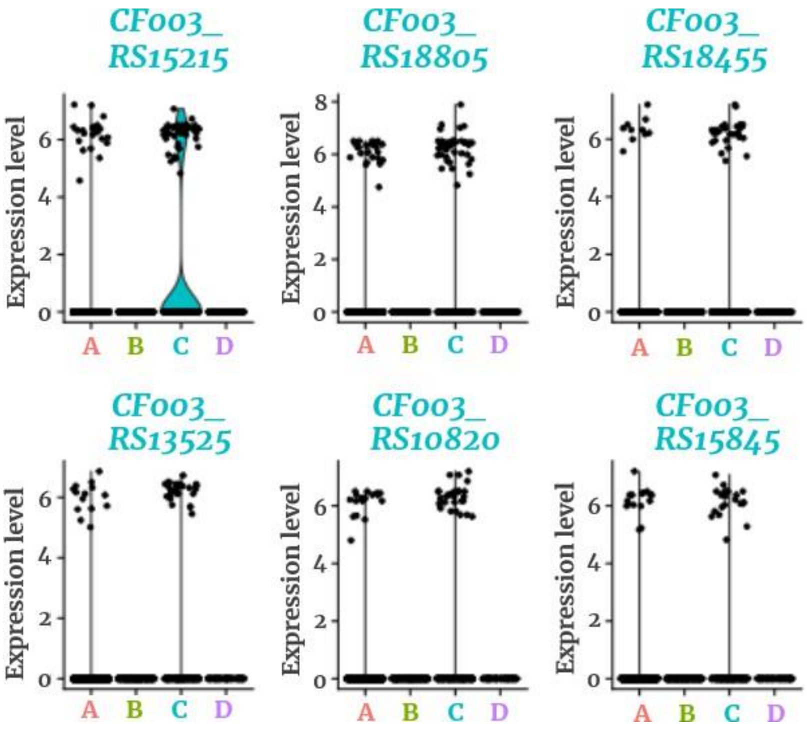

Figure S2. Expression distribution of sub-cluster C marker genes. Violin plots illustrate the expression levels of six marker genes specifically enriched in sub-cluster C.

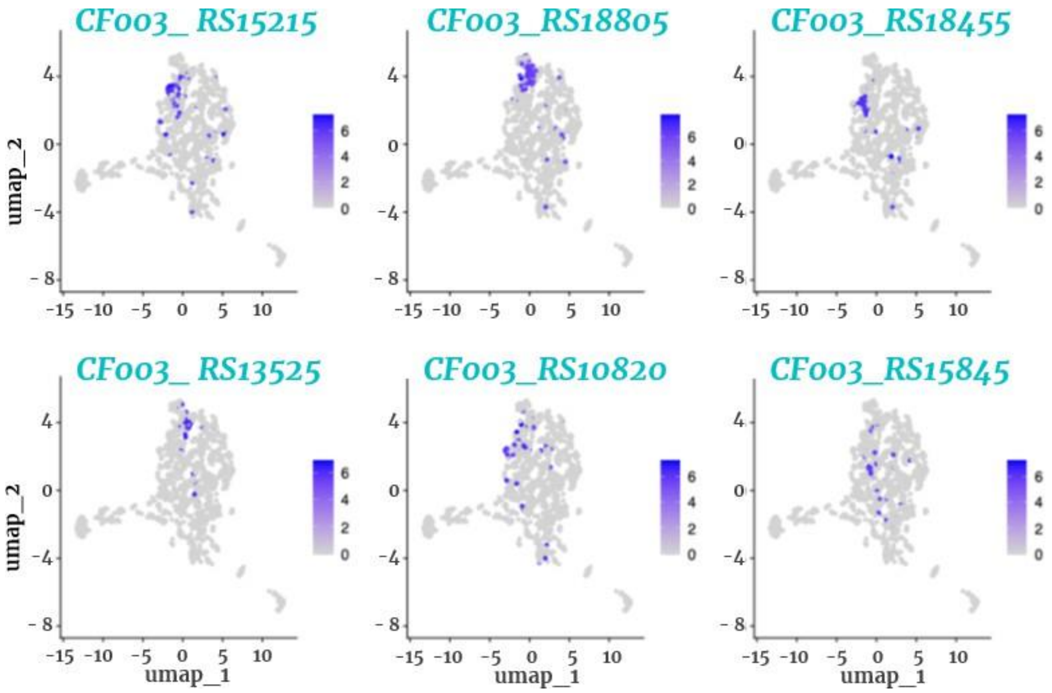

Figure S3. Spatial distribution of sub-cluster C marker genes in *P. gingivalis*. Feature plots displaying the expression patterns of six marker genes on the UMAP projection. Each panel represents a differentially expressed gene, with color intensity indicating its expression level within individual cells.
